# Effect of three LED lights on the biomass production and copper remediation by shoot cultures of *Musa paradisiaca*

**DOI:** 10.1016/j.heliyon.2020.e04981

**Published:** 2020-10-07

**Authors:** Tjie Kok, Fenny Irawati

**Affiliations:** Faculty of Biotechnology, University of Surabaya, Indonesia

**Keywords:** Environmental science, White, red, and blue LED lights, *Musa paradisiaca*, Copper remediation, Biomass production, Growth index

## Abstract

In photosynthesis, certain wavelengths of radiation are absorbed by photosynthetic pigments in plants, depending on the molecular structures of the pigments. Two main radiation wavelengths absorbed by plants for photosynthesis and biomass production are in the range of red and blue lights. Therefore, in addition to white light, observation applying the red and blue lights in plant researches gain great interest. The aim of this project is to compare the effect of three LED lights on the biomass production and copper remediation by previously-screened shoot cultures of *Musa paradisiaca.* The LED lights applied are arrangements of white, red, and blue LEDs, each arrangement having the same voltage of 12 V and the same power of 24 Watts. The shoot cultures were cultivated aseptically in 25 mL Murashige and Skoog agar media supplemented with 5 mg/L copper ions. Both parameters –the biomass production and copper concentration in biomass– determine the overall copper remediation by the shoot cultures from media. The results of this project show about 1.5-fold biomass production –expressed as growth index– was obtained upon the application of white light compared to that of the other two lights. Meanwhile, there is no significant difference in the concentration of copper in biomass upon the application of the three lights. Combined together, the application of white light still predominates for the overall copper remediation by the shoot cultures of *Musa paradisiaca* from media.

## 1 Introduction

Various contaminants in enviroment have been described as severely hazarding the health of living creatures [1]. Heavy metals, among them, are well known as dangerous contaminants because of their unbiodegradable properties. Hence they persist in environment for long period. Consequently, they may cause toxicity, even mutagenesis in living organisms. Biotechnology has employed plants for heavy metal remediation. The use of conventional plants for such purpose only uses variation of a species genetics to meet the characteristics required for remediation. Whereas, the elevation in the expression of certain protein or peptide in the plant's cells is expected to improve the remediation capacity of plants for metal ions [2, 3]. Such elevation could be achieved, among others, by plant-tissue-culture techniques. To this end, we need to modify the traits of plant candidates by certain treatments to produce more biomass and to improve their capability in removing metal ions from contaminated media.

The term “biomass” refers to the biological materials that can be used to achieve certain purpose, which in this case, is to remediate heavy metal ions from the growth media. This biomass could also be used to generate energy; and because the plants can be recultivated, then this source is a renewable source of biomass [4].

The commonly encountered problem when cultivating plant cultures in abnormal growth media, for example media containing heavy metals, is their low growth rate, resulting in the low biomass production. In our previous project, we have successfully screened shoot cultures of *Musa paradisiaca* that are able to grow in media containing copper ions with concentration up to 5 mg/L or 80 μM and capable in absorbing the ions from their growth media [5]. However, their capacity to remediate the ions from media was relatively low.

The use of artificial lights to improve crop production was made possible after the invention of robust and long-lasting electrical lamps in the beginning of twentieth century [6]. Nowadays, electrical lighting could be used as a steady and reliable source of radiation to control plant growth environment [7]. The use of LEDs gives higher lighting efficiency or lower energy consumption, over incandescent or compact fluorescent lamps [8, 9]. The red and blue LED alone or in combination has been applied for plant morphogenesis both in vivo and in vitro over the decades [10, 11, 12]. Light emitting diodes (LEDs) with customized color or wavelength could probably be applied on the plantlets to improve the biomass production [13, 14]. Hence the aim of this project is to apply red and blue LEDs –in addition to the white LED– on the previously-screened shoot cultures of *Musa paradisiaca* and compare the biomass production and overall copper remediation from growth media.

To this end, we made arrangements of white, red, and blue LEDs and apply them to the previously-screened shoot cultures of *Musa paradisiaca* cultivated on media containing copper ions of 5 mg/L. White LED, in this project, is used as a control. All the light arrangements are of the same voltage of 12 V and the same power of 24 Watts. The biomass production and the copper concentration in biomass of the shoot cultures are evaluated after 30-day incubation. The former is measured and expressed as growth index, that is the ratio of the fresh weight of harvested biomass to that of the initially-cultivated biomass. The latter is measured and expressed as milligram of copper per gram of dried biomass.

It is expected that having the greater growth index and/or increased concentration of copper in biomass, the selected group of shoot cultures of *Musa paradisiaca* could further be grown to produce plants having potential to remediate copper ions in polluted soil.

## 2 Materials and methods

All chemicals were purchased from Sigma-Aldrich, Indonesia. The lamps used were LEDs Strip Light SMD-12V IP 44, 24 W, Krisbow, Cina, consisting of white, red, and blue color (the wavelengths are not specified). The inductively couple plasma (ICP) used was ICAP 6200 Duo ICP-OES Spectrometer s/n: IC2D20124114, Thermo Scientific, UK. Shoot cultures were cultivated aseptically in 25 mL Murashige and Skoog (MS) agar media supplemented with 5 mg/L copper ions [5]. The cultures were grouped depending on the three LED lights applied, i.e. white, red, and blue. Each group consisted of 18 shoot cultures. The individual weight of fresh shoot culture, between 450 mg and 600 mg, was properly measured and was recorded as the initial weight of each. They were then incubated for 30 days and 14 out of 18 shoot cultures, that grew well and not contaminated, were harvested for subsequent steps. Harvest was conducted by first rinsing the shoot cultures with distilled water to free their surface from adsorbed media. The water was then allowed to evaporate on flowing air. The weight of individual harvested shoot culture was measured. The growth index was calculated as a ratio of the weight of each shoot culture at harvest time to the initial weight of it at time of cultivation. After drying in an oven and pulverizing the dried biomass, ± 10 mg of individual sample was destructed with 7.5 mL HNO_3_ 2%, sonicated for 10 min and filtrated with Whatman filter paper of 0.2 μm. The concentration of copper ions in filtrate was measured using ICP at wavelength of 324.75 nm [15]. The data was analyzed using SPSS V27.

## 3 Results

### 3.1 *Musa paradisiaca* shoot cultures and their growth indexes

**Figure 1:**
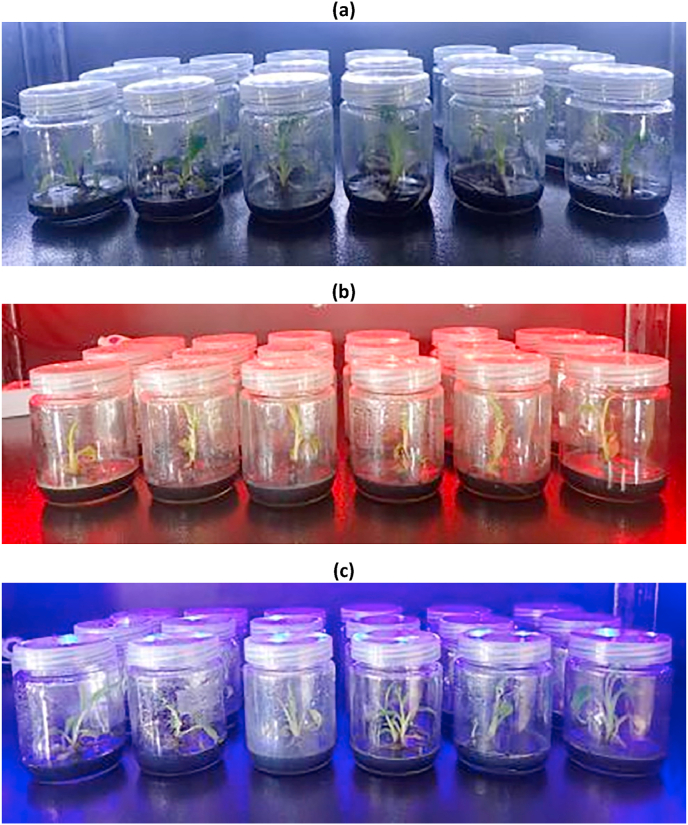
*Musa paradisiaca* shoot cultures. **(a)** White LED is applied **(b)** Red LED is applied **(c)** Blue LED is applied.

**Figure 2:**
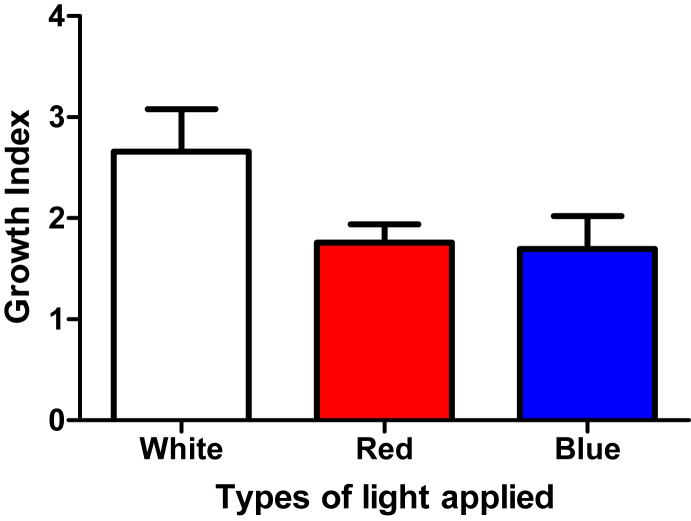
Biomass production expressed as growth index of each group of *Musa paradisiaca* shoot cultures upon the application of three LED lights (n = 14 for each group).

The shoot cultures of *Musa paradisiaca* cultivated in MS agar media supplemented with 5 mg/L copper ions [5] with the application of white LED (Figure 1a) produced the greatest growth index (expressed as mean ± SE), 2.658 ± 0.419 (Table 1 and Figure 2). Whereas, the ones with application of red and blue LEDs (Figure 1b and 1c) gave relatively the same growth index, 1.758 ± 0.182 and 1.696 ± 0.326 (Table 1 and Figure 2).

**Table 1:** Biomass production expressed as growth index of each group of *Musa paradisiaca* shoot cultures upon the application of three LED lights (n = 14 for each group).

Type of LED light applied	Growth index∗
White	2.658 ± 0.419
Red	1.758 ± 0.182
Blue	1.696 ± 0.326

∗ expressed as mean ± SE.

### 3.2 Copper concentration in dried biomass of *Musa paradisiaca* shoot cultures

**Figure 3:**
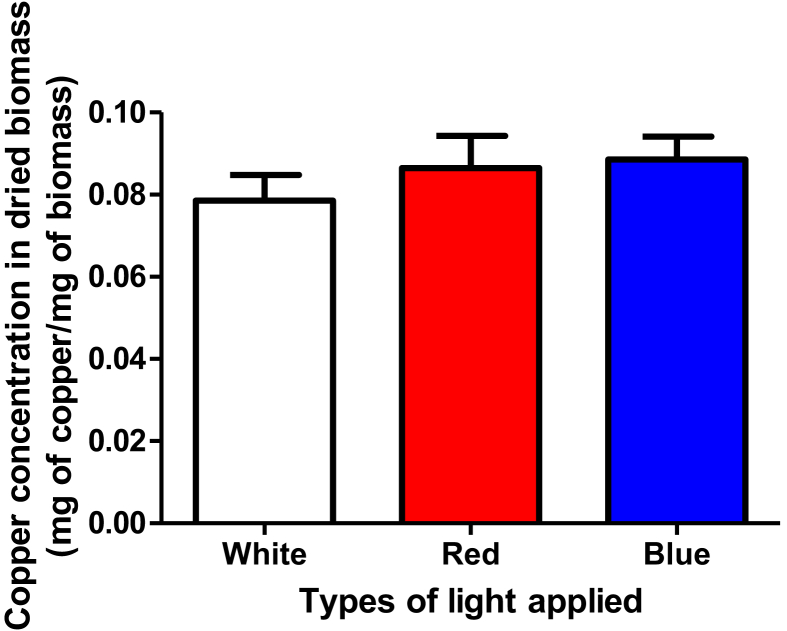
Copper concentration in the dried biomass of each group of *Musa paradisiaca* shoot cultures upon the application of three LED lights (n = 14 for each group).

The copper concentration in dried biomass of the group of shoot cultures of *Musa paradisiaca* irradiated with white LED (expressed as mean ± SE) is 0.079 ± 0.006 mg of copper/mg of biomass, as compared to 0.086 ± 0.008 mg of copper/mg of biomass and 0.089 ± 0.006 mg of copper/mg of biomass, respectively, of those irradiated with red and blue LEDs (Table 2 and Figure 3).

**Table 2:** Copper concentration in dried biomass of each group of *Musa paradisiaca* shoot cultures upon the application of three LED lights (n = 14 for each group).

Type of LED light applied	Copper concentration in dried biomass (mg of copper/mg of biomass)∗
White	0.079 ± 0.006
Red	0.086 ± 0.008
Blue	0.089 ± 0.006

∗ expressed as mean ± SE.

## 4 Discussion

The previously-screened shoot cultures of *Musa paradisiaca* were cultivated in 25 mL MS agar media supplemented with 5 mg/L copper ions [5] and irradiated with white, red, and blue LEDs. They all grew well (Figure 1) with reasonable growth indexes (Table 1 and Figure 2). In this project, we expected the group of shoot cultures irradiated with the customized wavelengths of radiation would yield overally greater remediation of copper ions from media.

The results of the project show that the group of *Musa paradisiaca* shoot cultures with the application of white LED produced growth index of 2.658 ± 0.419. Meanwhile, the ones irradiated with red and blue LEDs yielded growth index of 1.758 ± 0.182 and 1.696 ± 0.326, respectively. The analysis of varian –showing P < 0.05 followed by Duncan post hoc test– reveal that the growth index of the group of shoot cultures irradiated with white LED differs significantly, ca. 1.5-fold higher, from the one of the groups irradiated with red and blue LEDs. Meanwhile, the growth index of the group of cultures irradiated with red and blue LEDs are not different significantly from each other (Table 3). It may suggest that photosyntetic pigments of the shoot cultures enable the plants to select wavelengths of radiation suiting better their need for growth. The red and blue LEDs may suffer from the wavelength mismatch with the photosynthetic action spectrum. This can happen because even though the spectrum of LED is sufficiently narrow and it appears as a single color to human eyes, it is neither spectrally coherent nor highly monochromatic [9].

**Table 3:** Duncan post hoc test of growth index for each group of *Musa paradisiaca* shoot cultures upon the application of three LED lights (n = 14 for each group).

Type of LED light applied	Growth index
White	0.7700^a^ ± 0.006
Red	0.8314^a^ ± 0.008
Blue	1.8029^b^ ± 0.006

^a, b^The same symbols reperesents no real difference, the different ones represents a real difference.

Further, the results show that the group of *Musa paradisiaca* shoot cultures irradiated with white LED produced concentration of copper in dried biomass of 0.079 ± 0.006 mg of copper/mg of biomass, as compared to the one irradiated with red and blue LEDs, 0.086 ± 0.008 mg of copper/mg of biomass and 0.089 ± 0.006 mg of copper/mg of biomass, respectively (Table 2 and Figure 3). However, all the three are not significantly different from each other in analysis of varian (P < 0.05).

Combined together, the results show that application of white light still predominates over the red and blue lights for the project. Hence, we would rather apply white light that is more available on the market and cheaper rather than red and blue lights. This may also suggest that the application of customized wavelengths of radiation would not be really needed for such kind of work in plant tissue cultures.

## 5 Conclusion

The results of this project reveal that the application of white light still predominates over the red and blue lights for the overall copper remediation by the shoot cultures of *Musa paradisiaca*. Therefore, for such kind of work the application of white light would be more preferable than that of red and blue lights.

## Declarations

### Author contribution statement

Tjie Kok: Conceived and designed the experiments; Performed the experiments; Analyzed and interpreted the data; Contributed reagents, materials, analysis tools or data; Wrote the paper.

Fenny Irawati: Performed the experiments; Contributed reagents, materials, analysis tools or data.

### Funding statement

This work was supported by 10.13039/501100012696University of Surabaya, Indonesia (013/ST-Lit/LPPM-01/FTB/II/2019).

### Competing interest statement

The authors declare no conflict of interest.

### Additional information

No additional information is available for this paper.

## References

[1] Saxena G., Bharagava R.N. (2017). Organic and inorganic pollutants in industrial wastes. Environmental Pollutants and Their Bioremediation Approaches 23–56.

[2] Chandra R., Saxena G., Kumar V. (2015). Phytoremediation of environmental pollutants: an eco-sustainable green technology to environmental management.

[3] Chirakkara R.A., Cameselle C., Reddy K.R. (2016). Assessing the applicability of phytoremediation of soils with mixed organic and heavy metal contaminants. Rev. Environ. Sci. Biotechnol..

[4] Stephenson C., Black C.R. (2014). One step forward, two steps back: the evolution of phytoremediation into commercial technologies. Biosci. Horizons.

[5] Kok T. (2012). The accumulation of copper ions in biomass and its influence on the production of phytochelatins in shoot culture of Musa paradisiaca. International Conference on Chemical, Environmental and Biological Sciences (ICCEBS’2012) 38–40.

[6] Pinho P., Halonen L. (2017). Agricultural and horticultural lighting. Handbook Of Advanced Lighting Technology 703–720.

[7] Gupta S.D. (2017). Light Emitting Diodes for Agriculture: Smart Lighting.

[8] Zeb A., De Andrade Romero M., Baiguskarov D., Aitbayev S., Strelets K. (2016). LED lightbulbs as a source of electricity saving in buildings. MATEC Web Conf..

[9] Deng Y., Chu D. (2017). Coherence properties of different light sources and their effect on the image sharpness and speckle of holographic displays. Sci. Rep..

[10] Agarwal A., Gupta S.D. (2016). Impact of light-emitting diodes (LEDs) and its potential on plant growth and development in controlled-environment plant production system. Curr. Biotechnol..

[11] Gupta S.D., Jatothu B. (2013). Fundamentals and applications of light-emitting diodes (LEDs) in in vitro plant growth and morphogenesis. Plant Biotechnol. Rep..

[12] Naznin M.T., Lefsrud M., Gravel V., Hao X. (2016). Using different ratios of red and blue LEDs to improve the growth of strawberry plants. Acta Hortic..

[13] Korbee N., Figueroa F.L., Aguilera J. (2005). Effect of light quality on the accumulation of photosynthetic pigments, proteins and mycosporine-like amino acids in the red alga Porphyra leucosticta (Bangiales, Rhodophyta). Photochem. Photobiol..

[14] Yan C., Zhang L., Luo X., Zheng Z. (2013). Effects of various LED light qualities and light intensity supply strategies on purification of slurry from anaerobic digestion process by Chlorella vulgaris. Int. Biodeterior. Biodegrad..

[15] Thompson M., Walsh J.N. (1989). Analysis of metals by ICP-AES. Handbook of Inductively Coupled Plasma Spectrometry.

